# The Impact of Scholarly Concentration Programs on Graduates’ Career Choices and Interest in the Care of Older Patients

**DOI:** 10.7759/cureus.51697

**Published:** 2024-01-05

**Authors:** Lindsay Wilson, Casey Kelley, C. Ray Cheever, Elizabeth Harlow, Gwendolen Buhr

**Affiliations:** 1 Department of Medicine, Division of Geriatrics, University of North Carolina at Chapel Hill School of Medicine, Chapel Hill, USA; 2 Geriatrics, University of Nebraska Medical Center, Omaha, USA; 3 Geriatrics, Duke University Medical Center, Durham, USA

**Keywords:** elderly care, mentorship, professional identity formation, medical education, geriatrics, scholarly concentration programs

## Abstract

Background: More geriatricians are needed to care for the aging population. Geriatric scholarly concentration programs (GSCPs) may promote medical students’ interest in this underserved field or careers working with older adults. Additionally, graduates of GSCPs may be more comfortable and competent in providing care for older adults. Surveys were administered to graduates of GSCPs to determine the role of these programs in shaping medical students’ careers and views about caring for older adults.

Methods: The purpose of this study is to understand the impact of GSCPs on medical graduates’ career choices and self-perceived skill and comfort in caring for older adults. A Qualtrics survey (Qualtrics International Inc., Seattle, Washington, United States) was developed and distributed to 83 graduates of four GSCPs in the United States. Data were analyzed using a significance level of p>0.05 for all tests. Descriptive statistics were calculated to summarize the data. Wilcoxon signed-rank tests were used to test for significant differences in interest in pursuing a career in geriatrics or working with older adults. Qualitative responses were coded and analyzed for themes.

Results: A total of 34 out of 83 surveyed graduates of GSCPs indicated a higher interest in geriatrics as a career as well as increased comfort and self-perceived skill in caring for older adults after completing the GSCP. The components of the GSCP that most strongly improved the participants’ ability to care for older adults included the curriculum (n=31, 91%) and mentoring (n=28, 82%). An overwhelming majority of survey participants felt GSCPs should be offered as part of medical school programming (n=33, 97%).

Conclusion: This study suggests that GSCPs increase interest and competence in caring for older adults and increase interest in a career in geriatrics. GSCPs should be implemented across medical schools.

## Introduction

By 2030, all 73 million baby boomers will be over the age of 65 [1]. As the population ages, preparing physicians for the care of older adults becomes increasingly critical. Despite this growing need, the American Association of Medical Colleges (AAMC) does not mandate that all medical students meet the minimal geriatric competencies set forth by experts [2], and geriatric fellowship spots remain significantly unfilled [3]. 

To provide medical students with opportunities to individualize their training, nine United States medical schools have created geriatric scholarly concentration programs (GSCPs) that offer supplemental training and support for medical students interested in the care of older adults [4-6]. There is limited data regarding the efficacy of these programs in maintaining student interest in careers with older patients and preparing students for residency and clinical practice [6]. 

For example, at the University of North Carolina at Chapel Hill School of Medicine (UNC SOM), a GSCP entitled “Care of the Older Patient” was created in 2016 in response to a significant revision of the medical school curriculum. In line with national trends and the goal of providing more individualized learning, the preclinical curriculum at UNC SOM was shortened from 24 months to 18 months, resulting in the removal of a two-day required geriatrics course. Simultaneously, school leadership encouraged the development of scholarly concentration programs to support students’ unique career interests and educational goals. To preserve learning opportunities in geriatrics, educators in geriatrics responded by packaging existing elective courses in geriatrics and the ongoing National Institutes of Health (NIH)-supported summer research program, Medical Student in Aging Research (MSTAR), into the Care of the Older Adult Scholarly Concentration Program. 

Interested students applied with a letter and curriculum vitae, and all were accepted into the program. Students were assigned a geriatrician mentor with whom they met at least annually, and students were required to complete one geriatrics elective course or experience each year. For example, during their preclinical years, first-year medical students could take a semester-long service-learning course that required them to create an educational presentation for a community of older adults [7]. Outside of the required coursework, GSCP leadership at UNC sponsored social events and journal clubs to promote a sense of community among GSCP participants. Finally, students were expected to complete a scholarly project by graduation. 

All GSCPs share similar structural components to the one at UNC, consisting of longitudinal curricula, career mentorship, research opportunities, clinical opportunities, and community-building events [6]. Positive interactions with role models, safe spaces to "try on" a specialty, ample opportunities for reflection, shared stories and rituals, and informal networking all contribute to the development of professional identities [7]. One’s professional identity is how one “thinks of himself or herself as a doctor and is considered to be as critical to medical education as the acquisition of skills and knowledge relevant to patient care” [8]. 

A critical aspect of medical education is the contribution to trainees' professional identity formation. In fact, in 1957, thought leader Merton advised medical educators to instill in a trainee "a professional identity so that he comes to think, act, and feel like a physician" [9]. According to theory, professional identity formation is “mainly social and relational in nature” and “influenced more by the informal and hidden curriculum than formal teaching experiences” [10]. Experts at Stony Brook University, New York, emphasize "professional socialization" and curricula that foster positive relationships between students and role models as key to professional identity formation [11]. To this end, they recommend a gradual increase in social participation in the medical community, positive interactions with role models, and clinical experiences with patients and families [11].

Specific activities adopted by other institutions to foster this environment include interactive reflective writing, synergistic teaching modules about mindful clinical practices, strategies for effective use of a professional development e-portfolio, and faculty development in reflective coaching skills [12]. Other educators have developed curricula to target one facet of professional identity formation, such as teamwork [13]. Examples specific to teamwork include case-based learning and team-based learning activities [14].

Given that GSCPs offer role models, encourage relationships, foster dialogue, and sponsor social activities, they are likely to affect the professional identity formation of medical students in a way that promotes interest in the care of older adults. GSCPs may help students feel more comfortable and competent in their care of older patients and even encourage students to develop into doctors who pursue geriatrics or careers focused on working with older adults. 

## Materials and methods

This quantitative study utilized a survey designed to assess how GSCPs influenced medical students’ attitudes toward careers working with older adults and their career choices. A team with expertise in medical education and geriatric medicine developed the initial draft of the survey instrument based on study goals and experience working with medical students and scholarly concentration programs in geriatric medicine (see Appendix 1). They discussed what key stakeholders in medical education and geriatric medicine would want to know about GSCPs and formulated questions to obtain this knowledge. Next, survey questions were reviewed and edited by experts at the Odum Institute for Research in Social Science at UNC-Chapel Hill, whose purpose is to support research and offer consulting services in research methods. These experts suggested changes to the format to promote the reliability and validity of the survey design as well as ease of completion.

The survey was then pilot-tested by three medical students currently in the GSCP at UNC. Edits were made based on the students' feedback. The final version of the survey was then administered electronically, using Qualtrics Version XM (Qualtrics International Inc., Provo, Utah, United States) to graduates from four GSCPs. The leaders of each of the four GSCPs distributed the survey via the roster of email addresses they had on record for the graduates. Each graduate received an anonymous survey link via email, and identifiable information was not collected (see Appendix 2 for the recruitment email).

The survey evaluated the GSCP by asking participants about the components of the GSCP that improved their ability to care for older adults, the components that impacted their career choices, and the components that should be removed. To obtain more detail about the impact of the GSCP, participants were asked open-ended questions such as in what ways, if any, the GSCP made a difference in their career path and the care of older adults.

This study was approved as exempt from full review by the Institutional Review Board at UNC-Chapel Hill (IRB# 22-2750) and was approved by the Institutional Review Board at the University of Cincinnati (IRB#2023-0206). 

Quantitative data analysis: descriptive

Data were analyzed using SAS Version 9.4 (SAS Institute Inc., Cary, North Carolina, United States) using a significance level of p>0.05 for all tests. Descriptive statistics, including frequencies, means, and standard deviations, were calculated to summarize the data. To test for significant differences in interest in pursuing a career in geriatrics or working with older adults, Wilcoxon signed-rank tests were used. 

Qualitative data analysis: thematic

Two open-ended survey questions were analyzed using inductive coding, which allowed for general themes to be inferred from specific responses. Two coders (LW, CJK), reviewed all responses and developed their independent codebooks. The coders met to review their independent codes, resolve discrepancies, and create one consensus codebook, which was used by both coders to code all responses to both open-ended questions. The coders met again to compare codes and reconcile any differences in coding. Responses were categorized into making the participant more or less likely to pursue a career in geriatrics and how the curriculum impacted their care of older adults. 

## Results

Thirty-four participants of GSCPs (out of 83 who were initially sent the survey) from four different schools across the country responded to the survey, representing a 41% response rate. The respondents predominately identified as female (n=22, 65%) and White (n=28, 82%); four (12%) were Asian. The majority were medical residents (n=18, 53%), although a quarter of participants reported being in active clinical practice. A total of 33 (97%) reported practicing in an urban or suburban setting.

Participants agreed that they have worked with older adults in their practice “often or most of the time” (4.4 on a 5-point Likert scale). They additionally reported having taught their colleagues about older adults “sometimes to often” (3.7) and completed administrative tasks relating to older adults “some of the time” (3.4). Less often (2.4), they conducted research regarding older adults. 

Most graduates agreed or strongly agreed that participating in the GSCP prepared them to recognize (4.4) and assess (4.2) depression, recognize (4.5) and assess (4.5) cognitive impairment, recognize (4.6) and assess (4.4) delirium, and recognize (4.6) and assess (4.5) inappropriate medications in older adults. Similarly, most participants agreed or strongly agreed that the program prepared them to manage older adults’ medications (4.1), prioritize healthcare based on a patient’s values and goals (4.7), address end-of-life care (4.4), and address issues with mobility (4.2). 

**Figure 1:**
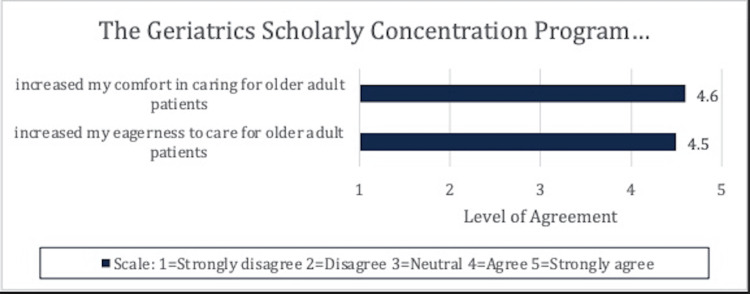
The GSCP increased comfort in caring for older patients and eagerness to care for older patients. GSCP: Geriatric scholarly concentration programs

**Figure 2:**
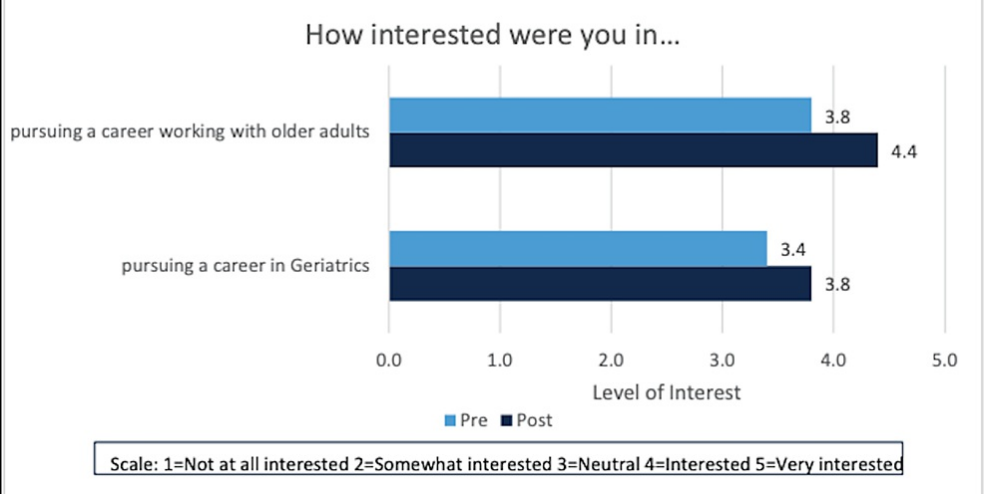
Increase in interest in careers working with older adults and pursuing a career in geriatrics before and after participation in the GSCP GSCP: Geriatric scholarly concentration programs

GSCPs were noted to increase both eagerness to care (4.5) and comfort in caring (4.6) for older adults (Figure 1). Participants noted an average “neutral to interested” interest in a career in geriatrics (3.4) or a career caring for older adults (3.8) prior to participation in the GSCP. At the conclusion of the program, a non-statistically significant shift was noted from 3.4 to 3.8 in interest in pursuing a career in geriatrics (Figure 2). A statistically significant change in interest in caring for older adults was noted (3.8 to 4.4, p<0.05). 

The components of the GSCP that most strongly improved the participants’ ability to care for older adults were curriculum (n=31, 91%) and mentoring (n=28, 82%). A total of 17 (50%) participants reported improvement due to community involvement and 35% (n=12) due to scholarly activity involved in the GSCP. The components that most impacted the career choices of the survey participants were mentoring (n=22, 65%) and curriculum (n=20, 59%), followed by community engagement (n=11, 32%) and scholarly project (n=8, 24%). Regarding participants’ views about the value of individual GSCP components, 12% (n=4) felt that the scholarly project could be removed, but the majority wanted all the components to continue (n=29, 85%). An overwhelming majority of survey participants (n=32, 94%) reported being very likely to recommend participation in a GSCP. Even more (n=33, 97%) reported feeling that such programs should be offered as part of medical school programing.

Of the 34 respondents, 19 answered the questions, “In what ways, if any, has the GSCP made a difference in your career path?” and “In what ways, if any, has the GSCP made a difference in your care of older adults?” Four of these (21%) credited the GSCP with affirming or furthering their decision to pursue geriatrics as a career. For example, one graduate stated that the program:

*“…greatly influenced my decision to pursue geriatric psychiatry…It is definitely one of the reasons I will be doing a geriatric psychiatry fellowship.”* 

Of the 19 (68%) responses, 13 indicated that the program had a positive influence on their future career paths despite not having led to the pursuit of a geriatrics fellowship. For example, one former student stated that s/he was:

“*More interested in affecting geriatric care policy within the emergency department*”. 

The respondents credited the GSCP with helping them to focus on patients’ goals of care/what matters most (n=5), recognizing and treating cognitive impairment (n=3), improving their bedside manner (n=2), and helping them to become more comfortable with working with older adults (n=10) (Table 1). 

**Table 1:** Responses to the question in what ways, if any, GSCP made a difference in their care of older patients GSCP: Geriatric scholarly concentration programs

Theme	Percentage of those who answered open-ended questions that addressed a particular theme	Representative Quotes
Goals of Care/ What Matters Most	26% (5/19)	“within the emergency department when it comes to geriatric patients. I've especially noticed that I focus heavily on asking questions regarding what a patient's goals are so that we do not do unnecessary testing/procedures/admissions. Changing from standard of care for every diagnosis/symptom to goals of care"; “learning that we can focus on their priorities rather than always following guideline directed medicine”
Recognizing/Treating Cognitive Impairment	16% (3/19)	“it helped start developing my approach to dementia assessments. I have continued to use some of the skills that I learned and saw modeled in attendings.”
Demeanor/Bedside Manner	11% (2/19)	“I am more caring and compassionate as a result of the perspective I gained being part of the GSCP.”
Comfort Working with Older Adults	53% (10/19)	“I feel much more comfortable recognizing and managing medications of older adults as well as post-operative care of older patients"; “… as an anesthesiologist, recognizing the adverse long term effects of anesthesia on older adults related to cognitive impairment, etc.”

## Discussion

GSCPs appear to be a feasible way to encourage interest in and improve the quality of care for older adults. Graduates of GSCPs agreed that the program enabled them to be more prepared to appropriately manage care for older adults across the 4Ms of geriatric medicine (mentation, mobility, medications, and what matters). 

GSCPs differ across the country in some components, but all GSCPs include mentorship, research, and additional geriatrics curriculum [6]. The specific curricular components and mentoring were the most important factors impacting career choices as well as the participants’ abilities to care for older adults. Nevertheless, the overwhelming majority of responders wanted the entire GSCP to continue, suggesting that the multi-faceted nature of these programs has value and should be maintained when possible. 

The primary goal of GSCPs is to improve medical students’ knowledge and appreciation for geriatric medicine and the older adults whom we serve. Routinely, students in GSCPs choose to pursue a myriad of fields (in both surgical and medical specialties), most commonly outside of geriatrics, with a few pursuing geriatrics [6]. This data supports the conclusion that concentrated exposure to geriatrics in a programmatic fashion during medical school not only increases interest and competence in caring for older adults but also shows a trend toward increasing interest in a career in geriatric medicine. 

There are limitations to this study due to survey and response bias. Graduates possibly felt inclined to answer in ways that more positively appraised the value of GSCPs, knowing that the survey was assessing the impact of these programs. The participants who responded were also likely those who had rewarding experiences and felt the GSCP positively impacted their career and education. The non-responders may have very different perspectives that the study unfortunately did not capture. Alternatively, non-responders may have held similar views but were too busy as practicing physicians to complete the survey. The survey response rate of 41% is consistent with a study that showed a physician survey response rate of 35% [15]. The data obtained from this study support our conclusions that GSCPs provide valuable education while supporting medical students’ interests in careers caring for older adults. 

As a result, there are practical implications from this study. GSCPs should be further explored as a viable approach to ensuring a trained physician workforce for our aging population. For example, further research could compare students who participated in GSCPs versus students who did not. With more evidence to support the efficacy of GSCPs, medical schools without GSCPS may invest in developing and implementing a GSCP to support student interest and competence in the care of older adults. Medical schools with GSCPs may then lobby for financial support and seek creative solutions to overcome any barriers to their operation. For example, at UNC-Chapel Hill, to address the lack of funding for faculty leaders, student leaders in the GSCP were identified and named Geriatric Medical Student Chiefs in July of 2022. They created service and clinical opportunities, hosted social events, recruited students, and assisted with the curriculum. Student recruitment of first-year medical students into the GSCP doubled. Potential next steps include evaluating this leadership model and other creative approaches that sustain GSCPs. 

## Conclusions

GSCPs may increase medical students’ interest and skill in caring for older adults and interest in careers in geriatrics. Therefore, GSCPs may serve a vital role in preparing medical students for an aging population, and GSCPs should be considered a potential strategy among efforts to develop a competent, caring, and age-friendly workforce. 

## Appendices

Appendix 2. Survey 

Demographic Information (what to Include?):

Current level of training or position: 

Resident IM, FM, Med-Peds 

Resident (Other specialty: write in:) 

Fellow Geriatrics 

Fellow (Other specialty; write in:) 

Practicing Physician IM, FM, Med-Peds, Geriatrics, (Other specialty; write in:) 

How much of your job is  

• teaching about older patients 

• working with older patients 

• doing research on older patients 

• administration related to older patients

Retrospective Pre-Post Questions

LIKERT: (not at all interested, somewhat interested, interested, very interested) 

How interested were you in pursuing Geriatrics as a career before participation in the Scholarly Concentration Program? 

How interested were you in pursuing Geriatrics as a career after participation in the Scholarly Concentration Program? 

How interested were you in pursuing a career working with older adults before participation in the Scholarly Concentration Program? 

How interested were you in pursuing a career working with older adults after participation in the Scholarly Concentration Program? 

5 Ms

LIKERT:  (strongly disagree, disagree, agree, strongly agree) 

The Geriatrics Scholarly Concentration program prepared me for recognizing depression in older patients. 

The Geriatrics Scholarly Concentration program prepared me for recognizing depression in older patients. 

The Geriatrics Scholarly Concentration program prepared me for assessing cognitive impairment in older patients. 

The Geriatrics Scholarly Concentration program prepared me for recognizing delirium in older patients. 

The Geriatrics Scholarly Concentration program prepared me for assessing delirium in older patients. 

The Geriatrics Scholarly Concentration program prepared me for recognizing potentially inappropriate medications for older adults. 

The Geriatrics Scholarly Concentration program prepared me for managing an older patient’s medications. 

The Geriatrics Scholarly Concentration program prepared me to be able to prioritize based on a patient’s healthcare values and goals. 

The Geriatrics Scholarly Concentration program prepared me for addressing end-of-life care for older patients. 

The Geriatrics Scholarly Concentration program prepared me for addressing mobility issues (ex. falls) in older adults. 

The Geriatrics Scholarly Concentration program increased my eagerness to care for older adult patients. 

The Geriatrics Scholarly Concentration program increased my comfort in caring for older adult patients. 

The Geriatrics Scholarly Concentration program prepared me for addressing mobility issues (ex. falls) in older adults. 

Curricular Components

Curriculum, Scholarly Project, Mentoring, Community Engagement (journal clubs, dinners, or other group activities)

Rank the following curricular components in order of impact on your ability to care for older patients: Curriculum, Scholarly Project, Mentoring, Community Engagement (journal clubs, dinners, or other group activities) 

Rank the following curricular components in order of impact on your career choice: Curriculum, Scholarly Project, Mentoring, Community Engagement (journal clubs, dinners, or other group activities) 

Which components would you keep: 

Pick all that apply:  

Curriculum, Scholarship, Mentorship, Community

Remove 

Pick all that apply:  

Curriculum, Scholarship, Mentorship, Community

What aspects of the Geriatrics Scholarly Concentration Program impacted your career choice?  Pick all that apply:  

Curriculum, Scholarship, Mentorship, Community 

What aspects of the Geriatrics Scholarly Concentration Program improved your ability to care for older patients?  Pick all that apply:  

Curriculum, Scholarship, Mentorship, Community 

LIKERT: (strongly disagree, disagree, agree, strongly agree) 

Participation in the curriculum of the Geriatrics Scholarly Concentration Program impacted my career choice. 

Participation in the scholarly project (research) of the Geriatrics Scholarly Concentration Program impacted my career choice. 

Participation in the mentorship of the Geriatrics Scholarly Concentration Program impacted my career choice. 

Participation in the community activities of the Geriatrics Scholarly Concentration Program impacted my career choice. 

What aspects of the Geriatrics Scholarly Concentration Program contributed to your ability to care for older patients?  Pick all that apply: Curriculum, Scholarship, Mentorship, Community 

LIKERT: (strongly disagree, disagree, agree, strongly agree) 

Participation in the curriculum of the Geriatrics Scholarly Concentration Program improved my ability to care for older patients. 

Participation in the scholarly project (research) of the Geriatrics Scholarly Concentration Program improved my ability to care for older patients. 

Participation in the mentorship of the Geriatrics Scholarly Concentration Program improved my ability to care for older patients. 

Participation in the community activities of the Geriatrics Scholarly Concentration Program improved my ability to care for older patients. 

Recommendation

Would you recommend participation in a Geriatrics scholarly concentration program? 

not at all, less likely, likely, very likely 

Would you recommend the development of a Geriatrics scholarly concentration program at a medical school? 

not at all, less likely, likely, very likely 

Feedback

What did you take away from your participation in a Geriatrics Scholarly Concentration program that you are using today? (free response) 

Appendix 1. Invitation to Participate 

Subject Line: [IMPORTANT] Help Us Improve Geriatrics Scholarly Concentration Programs: National Leaders in Geriatrics Education Are Requesting Your Feedback

Hello,  

This is a request for your participation in a research study “Evaluation of Geriatrics Scholarly Concentration Programs” (University of North Carolina Chapel Hill IRB# 22-2750).    

The purpose of this study is to learn more about the experiences and career outcomes of graduates of geriatrics scholarly concentration programs.  

We have identified you as someone who completed a geriatrics scholarly concentration program, and we would greatly appreciate your feedback on your experiences in the program.  

By completing this five-minute anonymous Qualtrics survey, you will be playing a major role in the design and implementation of geriatrics scholarly concentration programs for future medical students across the United States. We sincerely thank you for your time and participation.  

Your participation is fully voluntary and you may withdraw at any time.  To protect your identity as a research participant, answers will not be linked to your email and will be stored on a password-protected computer.   There are minimal risks to participation.  By clicking the link to the survey, you are consenting to participate.  This study has been found to be exempt by the UNC School of Medicine IRB. 

If you have questions or concerns about your rights as a research subject you may contact the UNC-CH Institutional Review Board at 919-966-3113 or . To contact the primary investigator, please email .

Thank you, 
